# DANGER! Crisis Health Workers at Risk

**DOI:** 10.3390/ijerph17155270

**Published:** 2020-07-22

**Authors:** Mason Harrell, Saranya A. Selvaraj, Mia Edgar

**Affiliations:** 1 School of Public Health, Harvard University, Boston, MA 02138, USA; 2 Independent Researcher, Woodstock, GA 20742, USA; saranya.selvaraj@gmail.com; 3 Independent Researcher, Honolulu, HI 96795, USA; miaedgar5890@gmail.com

**Keywords:** healthcare worker, health worker, occupational medicine, crisis, pandemic, epidemic, disaster, war, refugees, displaced persons

## Abstract

The occupational hazards of health workers (HWs) in standard work environments have been well defined in both the developed and developing world during routine working conditions. Less defined are the hazards to HWs during pandemics, epidemics, natural disasters, wars, conflicts, and other crises. How do crises affect the infrastructure of medical systems? What are the distinct needs of the patient population during crises? What are the peculiarities of the Crisis Health Worker (CHW)? What are the known CHWs’ occupational risks? What are the protective factors? By means of a PubMed search, we synthesized the most relevant publications to try to answer these questions. Failures of healthcare infrastructure and institutions include CHW shortages, insufficient medical supplies, medications, transportation, poorly paid health workers, security concerns, and the absence of firm guidance in health policy. Healthcare needs affecting the patient population and CHWs include crisis-induced injury and illness, hazardous exposures, communicable diseases, mental healthcare, and continuity of care for pre-crisis medical conditions. CHWs’ occupational hazards include supply deficiencies, infectious disease transmission, long working hours, staff shortages, financial reimbursements, mental fatigue, physical exhaustion, and inconsistent access to clean water, electricity, and Internet. CHWs suffer from injuries and illnesses that range from immediate, debilitating injuries to chronic, unforeseen effects like mental fatigue, physical exhaustion, anxiety, burnout, and even post-traumatic stress syndrome (PTSD). Protective factors include personal traits such as adaptability and resilience as well as skills learned through structured education and training. Success will be achieved by constructively collaborating with local authorities, local health workers, national military, foreign military, and aid organizations.

## 1. Introduction

Health workers (HWs) laboring during conditions of crisis (hereinafter, Crisis Health Workers or CHWs) might be thought of as being immune to injury or illness because their job is to heal the sick and injured. In reality, they are very vulnerable because they commonly prioritize their patients’ needs above their own.

Challenges faced during crises include increased needs of the population, direct damage on infrastructure affecting supplies and transportation, security concerns, absence of firm guidance in health policy, diversity of cultural backgrounds, and language barriers. Crisis-induced hazards exacerbate routine challenges in resource-low countries with poor infrastructure, endemic infectious disease transmission, long working hours, and staff shortages [1]. Whether the setting is resource-rich or resource-low, CHWs suffer mental fatigue, physical exhaustion, anxiety, burnout, and even post-traumatic stress syndrome (PTSD). The spectrum of injuries and illness can range from immediate, debilitating injuries to chronic, unforeseen effects. This paper provides an overview of and timeline of the hazards and injuries that CHWs face.

We searched through PubMed, Google Scholar, and the Uniformed Services University of the Health Sciences Learning Resource Center to retrieve publications and resources about challenges that CHWs endure. The free-text terms we used include: ‘healthcare worker,’ ‘deaths,’ ‘humanitarian crises,’ ‘mental health,’ ‘physical health,’ ‘violence,’ ‘infrastructure challenges during crises,’ ‘barriers to care during crises,’ ‘migrant,’ ‘refugee,’ ‘personality traits,’ ‘pandemic,’ ‘disaster preparedness for healthcare workers,’ ‘resilience,’ ‘acute stress disorder,’ ‘PTSD,’ ‘depression,’ ‘burnout,’ ‘anxiety,’ ‘Ebola,’ ‘Marburg Virus,’ ‘MERS,’ ‘covid-19,’ ‘SARS,’ ‘radiation’, ‘earthquake’, ‘WHO,’ ‘DMAT,’ ‘NGO,’ ‘de-skilling,’ ‘mental hazard,’ ‘physical hazard,’ and ‘infectious risk.’ Included references were drawn primarily from academic literature on crisis health workers published after 1980. We also included select organizational guidelines and studies on health workers in non-crisis settings and on humanitarian/relief workers in general to provide context and fill in gaps in literature on crisis health workers.

## 2. Effects of Medical System Infrastructure and Patient Populations on CHWs

Table 1 is a synthesis of publications that provides an overview of the infrastructure/system challenges facing CHWs.

Crises can be categorized by the burden on healthcare organizations. A small-scale mass casualty incident may be limited to a transient surge that typically affects only hospitals while a large-scale crisis can overwhelm an entire system, especially in settings where healthcare systems already have poor infrastructure. These infrastructure challenges contribute to hazardous working conditions faced by CHW.

In crisis settings, limited access to potable water, electricity, and the Internet that affect the general population also affect CHWs [2,4,6]. Lack of a reliable water source can lead to nosocomial infections and contribute to illnesses such as diarrhea that affect CHW health [2,6]. Electricity loss can be a serious challenge, especially in the event that the local healthcare system was dependent on electricity. Internet connectivity not only provides CHWs with medical resources for real-time referencing but also serves as a primary form of efficient communication, which is paramount during the early phases post-crisis and should be considered to be a top priority in the immediate aftermath of a disaster [4].

Infrastructure deficits specific to healthcare systems during a crisis includes logistical or physical collapse of healthcare infrastructure and institutions, insufficient supplies and medications, exodus of local health workers including specialists, and poor pay for the remaining health workers [2,5,7]. These deficits challenge CHW to provide care safely for patients and for themselves. It is difficult to maintain human resources in crisis work. In low-resource settings, there is often pre-existing scarcity of HWs with migration away from low-resource countries exacerbating the global shortage of HWs in these countries. Reasons for HW migration include low wages, political instability, poor socioeconomic conditions, brutal regimes, internal and regional armed conflicts, wars, fractionalization, employment in fields other than those of their expertise, frustration of practicing under institutional regulations of their native country, and proximity and historical links to larger and more developed countries. The migration of highly skilled professionals or intelligent people away from a particular country is a phenomenon coined ‘brain drain’ [9].

Humanitarian crises can be an impetus for further ‘brain drain.’ After the Haiti earthquake, local CHWs noted disenfranchisement and competition among healthcare facilities due to a lack of coordination between NGO and locally-based facilities. Local CHWs not employed by NGOs had difficulty attracting patients and sustaining their budgets as they faced competition from aid agencies offering free care. An internal ‘brain drain’ also occurred since local physicians were being pulled to work, often at higher salaries, for these NGOs. These barriers made it difficult for local CHWs and health systems to provide quality care [5].

When personnel are available, whether locally or through external aid organizations, their training may not always line up with their expected duties in the field. One study reviewing medical responses to earthquakes noted that the main personnel deficiency was due to a shortage of nursing staff, with nurse: physician ratios of 1:7 to 1:2, when most hospital commanders preferred a ratio of 2:1 or 1:1. Due to uneven staffing and variable patient caseloads, CHWs frequently had to practice outside their usual scope of care [3]. More recently, during the ongoing COVID-19 pandemic, which resulted in patients overwhelming hospitals past existing capacity, governments waived or accelerated the licensing processes because of HW shortages [10]. This brings to the forefront the need to standardize and un-complicate often cumbersome licensing and credentialing processes both internal to countries and globally. Care must be taken, however, to ensure that unqualified individuals do not end up in roles best filled by HWs. Recently, this was brought to light with Renee Bach, a 30-year-old American with no medical training, who ran a center for malnourished Ugandan children. She is being sued in Ugandan civil court over the deaths of 105 children who were treated at the critical care center she ran in Uganda [11].

Distinct healthcare needs of the patient population include healthcare for crisis-related health-hazardous effects, increased risk of communicable diseases, increased mental healthcare, continuity of care for pre-crisis medical conditions, and increased medical care for individuals with risk factors for severe illness from crisis-related health hazards (i.e., communicable disease). Populations during crises often include displaced populations. The terms ‘refugee’ and ‘migrant’ are often used interchangeably. While there is no formal definition of an international migrant, most agree a migrant is someone who changes his or her country irrespective of legal status. ’Immigrant’ is a term to define migrants who plan to take up permanent, legal residence in that country, while an asylum seeker is a person who has left their home country as a political refugee and is seeking residence in another country.

The migrant population is abundant with an estimated 1 billion worldwide migrants today of whom 258 million are international migrants and 763 million are internal migrants. 68 million of the world’s migrants are forcibly displaced. High-income countries have absorbed 64% of these displaced populations [12]. Forced migration is often distinguished by conflict-induced or crisis-induced displacement [13]. For refugees, legal status is generally the most important factor determining access to health services [8]. Specific challenges CHWs face when caring for migrants and refugees include language barriers and needing to rely on interpreters, adjusting to different cultural norms, limited access to standard healthcare interventions because of immigration status, and overcrowding and poor sanitation in refugee/migrant camps, which leads to increased risk of communicable, food, and waterborne diseases [2,8].

## 3. Crisis Health Workers’ Hazards

Table 2 is a synthesis of publications that provide an overview of common mental and physical hazards that crisis health workers experience while working in crisis situations.

Health workers volunteering for crisis relief are admirable for their dedication to care for others while often putting themselves in harm’s way. Health hazards affecting the patient population directly affect CHWs, and what is frequently overlooked is that CHWs also need health care and can become patients themselves. During the immediate aftermath of an acute crisis, a rescue phase is often chaotic and presents an increased risk of physical harm to responders. Some examples include physical hazards, psychological hazards causing trauma, targeted violence, and exposure to chemical, biological, radiological, nuclear, and explosive materials.

Responders to radiation incidents like Chernobyl and Fukushima are in the proximity of radiation or are treating patients exposed to ionizing radiation. This type of exposure, depending on the type of isotope, usually has little risk for CHWs. However, there might be long-term effects for personnel exposed to greater than 100 mSv (millisievert), which requires periodic follow-up health examinations [19].

Violence against CHWs is a well-established risk. CHWs have faced bombings, imprisonment, rape, torture, and execution/death [2,19,20]. During some conflicts, medical care has been considered as an act of resistance and a crime. In the ongoing Syria conflict, medical clinics and hospitals are targeted, 57% of hospitals have been damaged, and, through August 2019, 912 medical professionals have been killed [20].

Infectious risks include malaria, HIV, tuberculosis, leptospirosis, dengue, typhoid, H1N1, and parasites. Crises involving floods increase risks of water contamination with biological and chemical hazards, but many types of crises also lead to unreliable water supplies. Among crisis relief workers interviewed upon returning home from the 2010 Haiti earthquake, the 2004 Southeast Asian Tsunami, and the 2005 Hurricane Katrina in the US, the most frequently reported sickness was travelers’ diarrhea [21]. Unprotected CHWs involved with patient extraction/recovery work can develop bronchitis, asthma, rhinosinusitis, and aspergillosis from exposure to fungi, allergens, and irritants from moldy indoor environments following hurricanes, flooding, and tropical storms [22].

CHWs responding to outbreaks of infectious disease such as Ebola/Marburg virus or SARS-associated coronaviruses face the additional hazard of nosocomial infection. A recent systematic review from the *Journal of Infectious Disease* examined the number, frequency, and mortality of CHW infections and exposure risks in Ebola/Marburg virus outbreaks. CHWs were at increased risk of contracting infection compared to the lay population with insufficient/incorrect use of personal protective equipment (PPE) being the most frequently cited exposure risk. In many situations, deficiencies in the use of PPE arose from the lack of availability of appropriate equipment and/or the lack of training in PPE use during patient care, patient transport, cleaning, and environmental disinfection activities. Other factors contributing to CHW infection included inappropriate assessment/mis-triage, which results in exposure to patients with unrecognized infection, infrastructure deficiencies such as lack of running water or soap, inadequate hand hygiene, and lack of environmental/engineering controls such as isolation wards and standard infection prevention and control protocols [6].

In addition to the risk of becoming infected, health workers have reported anxiety associated with fear of contracting an infection. During the SARS outbreak of 2003, it was found that CHWs suffered from significant emotional distress associated with the fear of infection, quarantine, concern for family, perceived stigma from non-HWs, and the inability to refuse work assignments. These fears resulted in fatigue, insomnia, irritability, and decreased appetite. Almost half of those diagnosed with SARS infections were CHWs. Five of them died [17].

CHWs, who are exposed to seriously injured victims, dead bodies, and emotionally distressed victims, can develop burnout, psychological symptoms and disorders such as anxiety, depression, acute stress disorder (ASD), or even post-traumatic stress disorder (PTSD) [14,15,16]. According to Diagnostic and Statistical Manual of Mental Disorders (DSM) criteria, the diagnosis of ASD requires exposure to a traumatic event and three days to 30 days of at least one re-experiencing symptom, at least one avoidance symptom, at least two arousal and reactivity symptoms, and at least two cognition and mood symptoms [23]. Once these symptoms persist for over 30 days, ASD becomes PTSD. Fullerton et al. studied disaster workers from 9/11 and found that disaster workers with high exposure and previous disaster experience or who had ASD were more likely to develop PTSD [24]. A study of nurses following the 2008 Wenchuan Earthquake in China found that, one year after the earthquake, exposed nurses reported significantly more symptoms of PTSD (30.0%, *n* = 70) and depression (27%, *n* = 57) such as traumatic thought avoidance, intrusive thoughts, loss of pleasure, flashbacks, irritability, emotional numbing, and nightmares compared to non-exposed nurses [15].

Severe burnout is a three-part construct composed of exhaustion, cynicism, and a lack of professional efficacy. It is measured with the Maslach Burnout Inventory that covers these three areas: emotional exhaustion, depersonalization, and a low sense of personal accomplishment [25]. A longitudinal study of international humanitarian aid workers found that aid workers were at increased risk for depression and burnout (specifically the emotional exhaustion component of burnout) after returning from deployment, and this did not decrease after three to six months. There was also an increase in anxiety and burnout (depersonalization component) right after deployment and this decreased after three to six months [14].

At baseline, HWs have higher risk of chronic health conditions compared to the general population. A Korean study in 2014 investigated health insurance data for mental disorders in HWs and found a higher prevalence of mood, anxiety, sleep, and all psychiatric disorders compared to non-HWs [26]. Other studies have demonstrated an increased risk of peptic ulcer disease, metabolic syndrome, and cardiovascular disease in HWs compared to the general population, especially in association with stress, adverse psychosocial working conditions, and night-shift work [27,28,29].

Crisis settings expose CHWs to work settings with physical and psychological stressors that can increase the risk of chronic health conditions they are already predisposed to from routine work. In the aftermath of the 2004 Niigata, Japan earthquake, Azuma et al. studied how an increase in work hours affected cardiovascular risk factors by comparing disaster relief workers having the lowest workloads to those with the highest workloads. They found significantly greater increases in BMI, blood pressure, and serum cholesterol in staff with the highest workloads with the likely causes being increased stress, decreased sleeping time, and shift work. These changes persisted one year after the initial crisis [18]. Yip et al. studied the health burden among emergency medical service (EMS) workers and found that, 12 years after the 9/11 terrorist attack on the World Trade Center (WTC) in New York City, there was a high burden of health conditions associated with the WTC-exposure including depression (16.7%), PTSD (7%), harmful alcohol use (3%), rhinosinusitis (10.6%), gastroesophageal reflux disease (GERD) (12.1%), obstructive airway disease (11.8%), and cancer (3.1%) [16].

## 4. Crisis Health Workers’ Protective Factors

Table 3 is a synthesis of publications regarding protective factors for crisis health workers.

### 4.1. Helping CHWs Address Infrastructure Challenges

Professional training currently provided to CHWs does not uniformly address the occupational risks and scope of healthcare work rendered during a crisis. Williams et al. reviewed CHW crisis-training-effectiveness and found mixed evidence regarding training interventions improving objective measures. The current body of literature can still improve on definitive training protocols for CHWs [35]. Several studies, however, identify factors associated with improved CHW health outcomes while others highlight needs retrospectively identified by CHWs. These findings provide starting points for developing and testing interventions that will keep CHWs healthy and resilient.

In addressing health infrastructure challenges, it has been demonstrated in the literature that collaboration with local and international groups is an essential component of healthcare delivery success. In a review of four earthquakes in Turkey, Haiti, Armenia, and India, it was reported that collaboration of supplies, personnel, and transportation allowed for an increase in surge capacity, as field hospitals had highly trained personnel without logistical support and local liaisons were essential for patient evacuations [3]. A review of the effects of aid organizations in Haiti after the earthquake, however, found that, when local physicians were pulled to work for aid organizations, there was an increase in healthcare facility competition between NGOs and local facilities, which caused local facilities to close down. This exemplifies the need for collaboration between aid institutions and the local infrastructure that builds on and develops the latter [5].

Collaboration between aid institutions and local infrastructure are but one example of teamwork. Successful teamwork and the ability to work in a team are frequently cited factors in good outcomes for patients and CHWs. Principles of team-based healthcare in a standard healthcare setting include shared goals, clear roles, effective communication, measurable processes and outcomes, and leadership. This has been associated with improved job satisfaction for HWs, more efficient use of health-care services, and improved health outcomes [36].

According to the 2006 WHO report “Working together for health,” not only are large groups of volunteers needed, but skilled crisis coordination teams are essential [37]. Effective triage is critical because of the scarcity of skilled CHWs and an overwhelming demand during crises. Avoiding ‘de-skilling’ of highly trained staff is essential for building effective triages during emergencies. ‘De-skilling’ occurs when highly skilled workers are left to perform low-skilled work. HWs already working at high capacity under normal conditions are easily affected by the CHW migration during a crisis.

At the same time, despite the best attempts to plan otherwise, CHWs can find themselves working in roles different from what they expected or were trained to handle [2]. Returning to the comparison of responses to earthquakes in Armenia, Turkey, India, and Haiti, organizational adaptation was determined to be an essential component for success. Hospital structure and personnel allocation flexibility were rated as a high priority by all hospital commanders [3]. While switching roles can be important to a response’s success, this can also be a major source of stress for CHWs. Bjerneld et al. identified major categories of unexpected responsibility including leadership/managerial, communications, and teaching/pedagogic roles. One CHW reported having to calculate a hospital budget. Another was involved in a hospital renovation. A CHW reported expectations to “lead projects without having any previous training. It’s hard.” The interviewed CHWs identified additional coursework on technical topics such as public health, nutrition, tropical disease, and war surgery as contributing to successful missions. They also found a benefit from increasing their knowledge related to non-technical aspects of work such as education about leadership, leadership in another culture, teaching, foreign languages, and international aspects of development, conflict, law, and politics [2].

On the organizational level, CHWs found it helpful when NGOs deployed volunteers for appropriate lengths of time, closely supported first-time volunteers or paired them with experienced mentors, and provided adequate security checks and handoffs from prior teams. CHWs reported positive experiences with NGOs that provided accurate assignment descriptions and written materials with clear guidelines about organizational procedures and policies as well as instructions on managing situations outside their usual scope of practice [2].

### 4.2. Protecting CHWs from Harm

When it is impossible to eliminate a hazard, engineering controls that isolate CHWs from the hazard are the most effective means of hazard control. Attempting to manage behaviors (administrative controls) and personal protective equipment are less effective, even though all three methods (engineering, administrative controls, and personal protective equipment) should be employed to maintain CHW health and safety [38].

Addressing security concerns and physical infrastructure deficiencies such as lack of potable water or overcrowding can alleviate the risk of many acute physical harms affecting patients and CHWs in crisis settings [39]. Additional measures can be taken by leaders in local and international health organizations to reduce physical hazards of CHWs.

Implementation and adherence to policy and protocols have far-reaching effects. For example, prophylactic vaccination campaigns of CHWs are critical during epidemics, and strict PPE protocol implementation has been proven to be a key component of controlling epidemics [40]. CHWs saw reductions in the rate of illness and death from Ebola infection after implementing stringent infection control protocols [6]. Once strict PPE protocol was established and enforced during the 2014 MERS-Coronavirus outbreak, not only did infection rates of CHWs decrease, but anxiety decreased [33]. During infectious disease outbreaks and threats of bioterrorism, government policy can be critical to aid the rapid development of vaccines, antitoxins, and antimicrobials. This was highlighted during the 2001 anthrax cases [41].

Health hazards and injury risks can be influenced by past medical histories and chronic medical conditions, one reason to have pre-deployment physicals that include determining whether or not pre-existing medical conditions prohibit or limit CHW deployability. Pre-deployment physicals also provide a venue to optimize management of pre-existing conditions for vaccinating against communicable diseases and addressing the need for chemoprophylaxis treatment (i.e., malaria prophylaxis) before travelling to a crisis setting. Post-deployment healthcare can focus on screening for and addressing acute conditions as well as chronic conditions such as cardiovascular disease, gastroesophageal reflux disease, obstructive pulmonary disease, rhinosinusitis, depression, anxiety, and PTSD that affect CHWs. Post-deployment debriefings with a trained mental health specialist were valued by CHWs and should be considered an essential part of care for all CHWs [2].

### 4.3. Developing CHW Resilience

CHWs who actively volunteer to serve in crisis conditions must have mental strength, physical stamina, and emotional stability required to successfully perform technical skills under pressure in high-stress environments. Compared to other occupations, two distinguishing traits common to HWs are work drive—the disposition to work long, including irregular hours, and investing large amounts of time and mental acuity into work—and conscientiousness, which is defined as dependability, trustworthiness, and likeliness to adhere to standards. In addition to work drive and conscientiousness, other traits in HWs that correlate with more job satisfaction include optimism, emotional stability, assertiveness, extraversion, and teamwork [42]. Some of these traits, including emotional stability/self-care skills, dedication/motivation, and teamwork, were also identified as important to successful work in crisis settings [2,14,30,43].

When possible, recruitment of CHWs with these traits may increase success and safety during deployments. Experiences with crises are associated with the presence of protective qualities. However, this requires that the CHW first have experience [44]. Pre-deployment training programs can alternatively focus on developing these traits in all CHWs, such as by reviewing healthy coping strategies that promote self-care. Role-modeling also helps develop humanistic aspects of crisis work, which is similar to the apprenticeship style of healthcare training [34]. Training in leadership and teamwork can have the dual benefits of improving operation efficiency and reducing CHW stress [43]. Leadership traits developed during healthcare professional training and increased autonomy following such training can protect CHWs from negative psychological and physiologic consequences of stress [45].

Interviews of Canadian Disaster Medical Assistance Team (DMAT) members also identified additional core competencies required for disaster response that included a sense of flexibility, adaptability, and improvisation/innovation [30]. These traits may not be as inherent to HWs who, as noted earlier, are highly conscientious, and find success when adhering to standards and procedures when making clinical decisions [42]. In addition to providing CHWs with guidelines for practice in crisis settings, CHW training should focus on developing traits such as adaptability, improvisation, and austere medicine skills that might not be second nature for all HWs but can prepare CHWs for the novel stressors of crisis environments [30].

Many CHWs who have experience with crisis relief or work under austere conditions, however, never had time to prepare. They were simply doing their regular job when the crisis struck. In these situations, real-time interventions can help improve resilience and reduce stress and burnout. A pilot study of a psychoeducational intervention—Resilience and Coping for the Healthcare Community—showed that emphasizing team building, sharing experiences, and normalizing stressful feelings reduced stress levels in community health workers suddenly forced into the role of CHWs after Hurricane Sandy landed on the eastern coast of the United States in 2013. Coping strategies found to mitigate distress among CHWs included spending time with others by participating in activities that provide a sense of purpose, and asking for support when needed [32].

Resilience is critical for the successful CHW. It is defined as the capacity to recover quickly from difficulties, misfortune, or change. It is the process of adapting well in the face of adversity, trauma, tragedy, threats, or significant sources of stress. Ultimately, resilience is the nemesis of burnout. A four-year prospective follow-up study after the 2011 Japan Earthquake demonstrated that CHW resilience was characterized by vigor, dedication, and absorption. These traits were also associated with post-traumatic growth, defined as “the experience of positive change that occurs as a result of the struggle with highly challenging life crises.” Providing social support and spaces for deliberate reflection during and after crisis work could help CHWs exposed to traumatic situations follow a trajectory of post-traumatic growth that leads to increased work engagement in the future [31].

## 5. Conclusions

CHWs face numerous challenges that affect their health and well-being. Physical hazards arising as a consequence of crisis work often include exposure to the same threats affecting the patient population such as violence, radiation exposure, and infection. Some of these risks are specific to certain situations. Other risks, such as diarrheal disease, can arise from general infrastructure deficiencies common to a variety of crisis settings [21]. HWs are at risk for chronic illnesses and work in a crisis setting may increase their risk for a variety of chronic ailments including cardiovascular disease, obstructive airway disease, GERD, and rhinosinusitis [16,18].

Certain strategies can be used by CHWs and organizations to prepare for crisis work. If CHWs and their families both feel and are kept safe, they will be more available to work [33]. Technical training on protocols in managing exposure to radiation or infection, such as proper use of personal protective equipment and system-wide adherence to effective infection prevention and control guidelines, can reduce the risk of physical illness [6,33]. Pre-deployment/pre-employment physicals for workers in crisis settings provide the opportunity to screen for and optimize management of pre-existing illness, administer vaccinations, and prescribe chemoprophylaxis that reduces the risk of communicable diseases. Security checks can reassure CHWs that they are being supported in settings of military conflict [2].

Fear of physical harm is but one source of stress for CHWs. Healthcare during crises is challenging due to time-consuming collaborations with local authorities, international organizations, and NGOs. CHWs are required to simultaneously learn how to work with each other and to work with other organizations. Other sources of stress include witnessing traumatic events, heavy workloads, unexpected administrative and teaching duties, lack of clear guidelines, absence of mentorship/leadership, language/cultural barriers, and limited resources, which lead to substandard patient care [2,3,8]. Such stressors contribute to burnout, anxiety, depression, and PTSD in CHWs [14,15,16]. Traits such as a high level of motivation, flexibility, adaptability, emotional stability, self-care skills, ability to work in teams, and a multidisciplinary background characterize CHWs who are successful in meeting the challenges of crisis work. Aid organizations and other employers of CHWs should recruit individuals with these traits and design training programs to further cultivate these traits and develop CHW resilience. Psychological debriefings following crisis work can also help CHWs process trauma and facilitate a trajectory of growth following stressful experiences and should be considered essential [2,31].

In addition to interventions that develop stress-adaptive traits and coping strategies of individual CHWs, organizations can proactively reduce CHW stress by anticipating and addressing challenges such as language/cultural barriers, heavy workloads, and unexpected role-switching such as practicing outside of one’s scope of care or taking on non-clinical work. Heavy workloads and human resource shortages can be addressed by streamlining bureaucratic credentialing processes for CHWs, improved collaboration between local and international agencies, and proactively recruiting for positions known to be frequently short-staffed, such as nursing positions [3,11]. Clearly written guidelines and assignment descriptions should be provided by organizations in anticipation that CHWs will likely be compelled into roles not part of standard clinical training, such as setting up hospital infrastructure [2]. Training in public health and cultural competence should be offered regularly and tailored for specific circumstances. Organizational culture prioritizing a positive atmosphere and teamwork are also associated with increased CHW well-being [2,32,33].

Policy, positive personality traits, education, organizational culture, and training mitigate acute and chronic health risks. The above list, however, is only a starting point for keeping CHWs well. Literature on topics such as which methods of training are most effective for CHWs does not yet demonstrate one superior method [36]. The hierarchy of hazard controls, which is a well-accepted framework to prioritize hazard controls based on their effectiveness, emphasize engineering controls. These controls act on a system level and do not depend on modifications to an individual’s behavior [38]. Additional interventions will be identified and their effectiveness assessed only with more studies focusing on interventions that improve CHW well-being.

## Figures and Tables

**Table 1:** Medical system infrastructure and patient populations.

	Reference	Author (Year)	Relevant Terms
Barriers for overall crisis healthcare work	[2]	Bjerneld et al. (2004)	‘health system collapse,’ ‘armed conflicts,’ ‘isolation,’ ‘bureaucracy,’ ‘sexual discrimination’
	[3]	Bar-On et al. (2013)	‘earthquake,’ ‘nursing shortage,’ ‘early deployment,’ ‘variable patient caseload’
	[4]	Yamamura et al. (2014)	‘earthquake,’ ‘line disconnections,’ ‘loss of service,’ ‘satellite phones,’ ‘poor reception,’ ‘loss of Internet’
	[5]	Kligerman et al. (2017)	‘earthquake,’ ‘hospital collapse,’ ‘brain drain,’ ‘competition among healthcare facilities’
	[6]	Selvaraj et al. (2018)	‘Ebola outbreak,’ ‘lack of running water,’ ‘lack of electricity,’ ‘HW shortage’
	[7]	Suarez et al. (2019)	‘anthropogenic disaster,’ ‘insufficient medical supplies,’ ‘brain drain’
Specific barriers to care for migrants and refugees	[2]	Bjerneld et al. (2004)	‘communicating with interpreters,’ ‘different culture’
	[8]	WHO (2018)	‘irregular immigrants,’ ‘refugees,’ ‘legal status,’ ‘overcrowding,’ ‘poor sanitation,’ ‘medical risks for migrant populations’

**Table 2:** Crisis health workers’ hazards.

	Reference	Author (Year)	Relevant Terms
Mental Health Hazards	[14]	Lopes Cardozo et al. (2012)	‘depression,’ ‘burnout,’ ‘anxiety’
	[15]	Zhen et al. (2012)	‘depression,’ ‘post-traumatic stress disorder’
	[16]	Yip et al. (2016)	‘depression,’ ‘post-traumatic stress disorder,’ ‘harmful alcohol use’
Physical Health Hazards	[17]	Maunder et al. (2003)	‘SARS infection,’ ‘death,’ ‘fatigue,’ ‘insomnia’
	[2]	Bjerneld et al. (2004)	‘bombings,’ ‘mines,’ ‘crimes,’ ‘food shortage,’ ‘diarrhea,’ ‘weight loss’
	[18]	Azuma et al. (2012)	‘overwork,’ ‘hypertension,’ ‘hyperlipidemia,’ ‘elevated Body Mass Index (BMI)’
	[19]	Wolbarst et al. (2010)	‘ionizing radiation’
	[20]	Sibbald (2013)	‘imprisonment,’ ‘torture,’ ‘death’
	[21]	Costa et al. (2015)	‘violence,’ ‘accidents,’ ‘diarrhea,’ ‘vector-borne disease,’ ‘tuberculosis,’ ‘HIV,’ ‘hepatitis A,’ ‘leptospirosis,’ ‘typhoid fever,’ influenza,’ ‘malaria’
	[16]	Yip et al. (2016)	‘rhinosinusitis,’ ‘obstructive airway disease,’ ‘gastroesophageal reflux disease’
	[6]	Selvaraj et al. (2018)	‘Ebola virus infection,’ ‘Marburg virus infection,’ ‘death’

**Table 3:** Crisis health worker’s protective factors.

	Reference	Author (Year)	Relevant Terms
Protective Factors Individual	[2]	Bjerneld et al. (2004)	‘multidisciplinary background,’ ‘prepatory coursework,’ ‘team player,’ ‘emotional stability,’ ‘motivation,’ ‘language skills,’ ‘clinical experience,’ ‘field experience’
	[14]	Lopes Cardozo et al. (2012)	‘strong social network,’ ‘high level of motivation’
	[30]	Peller et al. (2013)	‘self-care skills,’ ‘survival skills,’ ‘flexibility,’ ‘adaptability,’ ‘critical thinking,’ ‘situational awareness,’ ‘problem solving,’ ‘leadership traits’
	[31]	Nishi et al. (2016)	‘resilience,’ ‘dedication,’ ‘post-traumatic growth’
	[32]	Powell et al. (2016)	‘healthy coping strategies,’ ‘normalizing/identifying stress responses’
Protective Factors Organizational	[2]	Bjerneld et al. (2004)	‘NGOs,’ ‘handovers,’ ‘written guidelines,’ ‘planning,’ ‘strong leadership,’ ‘security measures,’ ‘accurate assignment descriptions,’ ‘mentorship,’ ‘recruitment evaluations,’ ‘debriefing’
	[3]	Bar-On et al. (2013)	‘logistics self-sufficiency,’ ‘operational versatility,’ ‘collaboration between local and international teams’
	[30]	Peller et al. (2013)	‘teamwork,’ ‘leadership,’ ‘delegation,’ ‘communication,’ ‘education’
	[33]	Khalid et al. (2016)	‘positive atmosphere,’ ‘receiving appreciation/thanks,’ ‘personal protective equipment,’ ‘infection control guidance’
	[32]	Powell et al. (2016)	‘psychoeducation,’ ‘team building’
	[34]	Ren et al. (2017)	‘role modeling’
	[6]	Selvaraj et al. (2018)	‘personal protective equipment,’ ‘infection prevention and control,’ ‘triage’
